# Late Bilateral Sequential Cochlear Implant and Quality of Life

**DOI:** 10.1055/s-0043-1776721

**Published:** 2024-02-05

**Authors:** Larissa Claret De Lima Mendes, Alda Borges, Fernanda Caldas, Juliano Passos Barbosa, Fayez Bahmad Jr

**Affiliations:** 1 Department of Health Sciences, Faculty of the Universidade de Brasília, Universidade de Brasília, Brasília, DF, Brazil; 2 Department of Health Sciences, University of Brasilia, Campus Universitário Darcy Ribeiro, Brasília, DF, Brazil; 3 Department of Health Sciences, University of Brasília, Brasília, DF, Brazil; 4 Department of Medicine, Centro Universitário do Planalto Central Apparecido dos Santos, Brasília, DF, Brazil; 5 Health Sciences School, Universidade de Brasília, Brasilia, DF, Brazil

**Keywords:** quality of life, cochlear implant, hearing loss, questionnaire

## Abstract

**Introduction** Hearing impairment is one of the main disorders that can interfere with the development of speech and language. In an individual, it can cause significant communication difficulties, social isolation, negative feelings, and depressive disorders. The hearing aids (HAs) and cochlear implant (CI) are options for profound and severe hearing loss, and the CI can be indicated for individuals who do not obtain benefits from HAs.

**Objective** To evaluate the quality of life of individuals who underwent sequential bilateral CIs with a long surgical interval between procedures.

**Methods** Fifteen patients, aged 8 to 70 years old, who underwent sequential bilateral CI, with an interval ≥ 4 years between surgeries, were evaluated. Quality of life was evaluated using three questionnaires: WHOQOL-BREF, SSQ-12 and HHIA in Portuguese.

**Results** The WHOQOL-BREF questionnaire showed that the study participants had a good quality of life in all domains assessed. According to the SSQ-12, few reported inability to listen in communication situations. Most individuals were classified as having medium disability by the HHIA, but the social and emotional effects did not significantly affect the quality of life.

**Conclusion** The use of questionnaires to assess the quality of life of patients with hearing impairment is a valuable tool to measure adaptation to CI. Patients undergoing bilateral sequential CI, even with a long interval between procedures, presented high indices of quality of life.

## Introduction

Hearing impairment is one of the main disorders that can interfere with the development of speech and language. The Joint Committee on Infant Hearing 1 recommends a program for early detection and intervention of hearing, with hearing screening completed by the end of the 1 ^st^ month of life, complete audiological diagnosis in the 2 ^nd^ month of life, and early intervention in the 3 ^rd^ month of life. Hearing impairment in an individual can cause significant communication difficulties, social isolation, negative feelings, and depressive disorders. This is aggravated when hearing loss is severe and profound, which may compromise an individual's relationship with other people and their quality of life.

With the advancement of health technologies, humans can benefit from the technological resources available for enabling and rehabilitating hearing loss. The Hearing aid (HA) and cochlear implant (CI) are options for profound and severe hearing impairments, and the CI can be indicated for individuals who do not obtain benefits from HA. The CI is a biomedical, biocompatible, and durable electronic device developed to perform the functions of damaged or absent hair cells by transforming sound energy into low levels of electrical current and providing electrical stimulation of the remaining fibers of the auditory nerve. 2

However, even with the current innovative technology of CIs and how they enable users to understand speech in quiet environments, some difficulties arise in everyday situations, such as the location of sounds and understanding speech, especially in noisy environments. Such functions require the ability of binaurality, which may not be facilitated with the use of unilateral CIs. 3 The indication of bilateral CIs is an alternative to promote binaurality and has been implemented in recent years in the international context. 4

In Brazil, bilateral CI was included in health insurance plans, based on the Normative Resolution of the National Supplementary Health Agency (ANS, in the Portuguese acronym) RN No. 261 on July 28, 2011, and the Brazilian Unified Health System (SUS, in the Portuguese acronym), Ordinance no. 2,776/GM/MS, on December 18, 2014. Bilateral CI surgery can be performed in two ways: simultaneously or sequentially. In a simultaneous procedure, the two internal devices are inserted in a single surgical procedure; in a sequential procedure, the second internal device is implanted at another time, months or years after the first surgery. In cases of sequential surgery, researchers in the field have been discussing postsurgical results, especially the impact of the time interval between the first and second surgery on the speech recognition results. 5

The quality of life (QoL) is a term used both in everyday language by the general population and in scientific research in different fields of knowledge. In the area of health, the concept of QoL is recent and has been changing; the improvement in QoL has become an expected result of both care practices and public policies. 6 Quality of life measures can provide information on social and personal aspects, as well as measures physical and psychological well-being, incorporating the point of view of the patient.

Studies on quality of life may collaborate essentially to the areas of audiology and otology, more specifically with multidisciplinary teams in cochlear implants, to evidence the results of QoL in these patients measured methodologically. They may also help and confirm decision-making regarding the indication of cochlear implants, especially in patients undergoing sequential bilateral implants.

The objective of the present study was to evaluate the QoL of patients with sequential bilateral CI with an interval ≥ 4 years between surgeries through three questionnaires: World Health Organization Quality of Life (WHOQOL-BREF **)** , Speech, Spatial and Qualities of Hearing Scale (SSQ-12) and Hearing Handicap Inventory for Adults (HHIA) because there are no studies in the literature involving such questionnaires simultaneously and these are widely used in QoL studies. It is expected to quantify the social and emotional effects of hearing impairment, to evaluate subjective experiences and to quantify the listening skills in communication situations and, finally, to evaluate the QoL in the social, environmental, physical, and psychological spheres.

## Materials and Methods

This is a retrospective study. Data were collected after approval by the Research Ethics Committee of the School of Sciences and Health, under opinion no 4,327,050.

The sample consisted of 15 individuals, with ages ranging from 8 to 70 years old. The following inclusion criteria were considered: interval between the first and second CI surgery ≥ 4 years, regardless of patient age and time of first CI; medical and speech-language therapy postsurgery follow-up at the Cochlear Implant Center; the mental ability to understand and answer the questions (minors and the elderly were accompanied by at least one guardian during the administration of the questionnaires). The exclusion criteria were as follows: patients with other neurological pathologies that made it impossible for them to understand the questions and children < 6 years old due to immaturity in understanding and answering the questionnaires.

The evaluation of the QoL of the patients was performed using three questionnaires in Portuguese: WHOQOL-BREF, the SSQ-12, and the HHIA.

### World Health Organization Quality of Life (WHOQOL-BREF) Questionnaire

For the present study, the WHOQOL-BREF questionnaire ( Table 1 ) was selected for QoL assessment. The WHOQOL-BREF consists of 26 questions, of which 2 are general quality of life questions (general domain) and the remaining 24 questions represent each of the 24 facets that make up the original instrument (WHOQOL-100). Thus, the 24 questions cover 4 domains (Physical, Psychological, Social Relations, and Environment), and each facet is represented by a question, as shown in Table 2 .

**Table 1:** The WHOQOL-BREF questionnaire

Instructions:				
This questionnaire asks how you feel about your quality of life, health, and other areas of your life. Please answer all the questions. If you are unsure about which response to give to a question, please choose the one that appears most appropriate. This can be your first response.				
Please keep in mind your standards, hopes, pleasures and concerns. We ask that you think about your life in the **last two weeks** .				
*For example, thinking about the last two weeks, a question might be:*				
**How much do you worry about your health?**				
Not at all	A little	A moderate amount	Very much	An extreme amount
1	2	3	4	5
You should circle the number that best fits how much you have worried about your health over the last two weeks. So you would circle the number **4** if you worried about your health “ **very much** .”				
**How much do you worry about your health?**				
Not at all	A little	A moderate amount	Very much	An extreme amount
1	2	3	4	5
If you have worried “ **Not at all** ” about your health, you would circle number **1** . Please read each of the following questions, assess your feelings, and circle the number on the scale for each question that fits best for you.				

**Table:** 

** Please read the question, assess your feelings OVER THE LAST TWO WEEKS and circle the number on the scale for each question that gives the best answer for you. **						
		Very poor	Poor	Neither poor nor good	Good	Very good
1	How would you rate your quality of life?	1	2	3	4	5
		Very dissatisfied	Dissatisfied	Neither satisfied nor dissatisfied	Satisfied	Very satisfied
2	How satisfied are you with your health?	1	2	3	4	5
**The following questions ask about how much you have experienced certain things in the last two weeks.**						
		Not at all	A little	A moderate amount	Very much	An extreme amount
3	To what extent do you feel that physical pain prevents you from doing what you need to do?	1	2	3	4	5
4	How much do you need any medical treatment to function in your daily life?	1	2	3	4	5
5	How much do you enjoy life?	1	2	3	4	5
6	To what extent do you feel your life to be meaningful?	1	2	3	4	5
7	How well are you able to concentrate?	1	2	3	4	5
8	How safe do you feel in your daily life?	1	2	3	4	5
9	How healthy is your physical environment?	1	2	3	4	5
**The following questions ask about how completely you have experienced or were able to do certain things in the last two weeks. Circle your best answer number.**						
		Not at all	A little	A moderate amount	Very much	Extremely
10	Do you have enough energy for everyday life?	1	2	3	4	5
		Not at all	A little	A moderate amount	Very much	Extremely
11	Are you able to accept your body appearance?	1	2	3	4	5
12	Have you enough money to meet your needs?	1	2	3	4	5
13	How available to you is the information you need in your day-to-day life?	1	2	3	4	5
14	To what extent do you have the opportunity for leisure activities?	1	2	3	4	5
15	How well are you able to get around physically?	1	2	3	4	5
**The following questions ask about how good or satisfied you have felt about aspects of your life over the last two weeks.**						
		Very dissatisfied	Dissatisfied	Neither satisfied nor dissatisfied	Satisfied	Very satisfied
16	How satisfied are you with your sleep?	1	2	3	4	5
17	How satisfied are you with your ability to perform your daily living activities?	1	2	3	4	5
18	How satisfied are you with your capacity for work	1	2	3	4	5
19	How satisfied are you with yourself?	1	2	3	4	5
20	How satisfied are you with your personal relationships?	1	2	3	4	5
21	How satisfied are you with your sex life?	1	2	3	4	5
22	How satisfied are you with the support you get from your friends?	1	2	3	4	5
23	How satisfied are you with the conditions of your living place?	1	2	3	4	5
24	How satisfied are you with your access to health services?	1	2	3	4	5
25	How satisfied are you with your transport?	1	2	3	4	5
**The following question refers to how often you have felt or experienced certain things in the last two weeks.**						
		Never	Seldom	Quite often	Very often	Always
26	How often do you have negative feelings such as blue mood, despair, anxiety, or depression?	1	2	3	4	5

**Table 2:** Domains and QOL features WHOQOL-BREF

Domains	QOL Features
**Domain: Physical**	Pain and discomfort
	Energy and fatigue
	Sleep and rest Activities of Daily Living
	Dependence on medicinal substances and medical aids
	Work Capacity
**Domain: Psychological**	Positive feelings
	Negative feelings
	Thinking, learning, memory, and concentration
	Self-esteem
	Bodily image and appearance
	Spirituality/Religion/Personal beliefs
**Domain: Social relationships**	Personal relationships
	Social support
	Sexual activity
**Domain: Environment**	Financial resources
	Freedom, physical safety, and security
	Health and social care: accessibility and quality
	Home environment
	Opportunities for acquiring new information and skills
	Participation in and opportunities for recreation/leisure activities
	Physical environment (pollution/noise/traffic/climate)
	Transport
**Domain: General (Quality Self-Assessment)**	Quality of life and health perceptions

The WHOQOL-BREF questions have four types of response scales: intensity (ranging from nothing to intense), capacity (ranging from nothing to complete), frequency (ranging from never to always) and evaluation (ranging from very dissatisfied to very satisfied) and very poor to very good), all graded in five levels. The responses are scored one to five, and for questions 3, 4, and 26, the scores are inverted as a function of 1 = 5; 2 = 4; 3 = 3; 4 = 2 and 5 = 1. Table 3 shows the relationship of the 26 questions and the domains that each question represents, according to the syntax presented by Fleck et al.

**Table 3:** List of WHOQOL-BREF questions and their respective domains

Domain	Questions
Self-assessment of quality of life	1 and 2
Physical	3, 4, 10, 15, 16, 17, and 18
Psychological	5, 6, 7, 11, 19, and 26
Social relationships	20, 21 e 22
Environment	8, 9, 12, 13, 14, 23, 24, and 25

The questionnaire does not admit a total QoL score. The World Health Organization considers the premise that quality of life is multidimensional; therefore, each domain is scored separately. The questionnaire was administered one on one with each participant by an examiner.

### Speech, Spatial and Qualities of Hearing Scale (SSQ-12 *)* Questionnaire

This questionnaire aims to evaluate the hearing ability and quantify the listening skills in day-to-day communication situations. It includes a variety of domains, such as directional listening situations related to different distances, simultaneous voices, ease of listening, naturalness and clarity of everyday sounds. From this context, three general domains were identified, namely, hearing for speech, spatial hearing, and other auditory qualities.

The 12 questions that make up the SSQ-12 ( Table 4 ) are derived from version 5.6 of the SSQ-49 and address its main factors, including questions involving the 3 main domains, as well as 9 of the 10 pragmatic subscales (speech in silence, speech in noise, speech in speech, listening to multiple speech streams, location, distance and movement, segregation, sound identification, quality and naturalness and listening effort), considering the broader version of the SSQ-49 (49 items).

**Table 4:** Speech, Spatial and Qualities of Hearing Scale (SSQ-12) Questionnaire

SSQ49index	SSQ12index	Item	Pragmatic subscale
**1.1**	**1**	**You are talking with one other person and there is a TV on the same room. Without turning the TV down, can you follow what the person you are talking to says?**	**Speech in noise**
**1.10**	**2**	**You are listening to someone talking to you, while at the same time trying to follow the news on TV. Can you follow what both people are saying?**	**Multiple speech streams**
**1.11**	**3**	**You are in conversation with one person in a room where there are many other people talking. Can you follow what the person you are talking to is saying?**	**Speech in speech**
**1.4**	**4**	**You are in a group of about five people in a busy restaurant. You can see everyone else in the group. Can you follow the conversation?**	**Speech in noise**
**1.12**	**5**	**You are with a group, and the conversation switches from one person to another. Can you easily follow the conversation without missing the start of what each new speaker is saying?**	**Multiple speech streams**
**2.6**	**6**	**You are outside. A dog barks loudly. Can you tell immediately where it is, without having to look?**	**Localization**
**2.9**	**7**	**Can you tell how far away a bus or a truck is, from the sound?**	**Distance and movement**
**2.13**	**8**	**Can you tell from the sound whether a bus or truck is coming toward you or going away?**	**Distance and movement**
**3.2**	**9**	**When you hear more than one sound at a time, do you have the impression that it appears to be a single jumbled sound?**	**Segregation**
**3.7**	**10**	**When you listen to music, can you make out which instruments are playing?**	**Identification of sound**
**3.9**	**11**	**Do everyday sounds that you can hear easily seem clear to you (not blurred)?**	**Quality & Naturalness**
**3.14**	**12**	**Do you have to concentrate very much when listening to someone or something?**	**Listening effort**

Domains and pragmatic subscales derived from SSQ49. The SSQ12 is an abbreviated version that contains 12 items from the original.

### Hearing Handicap Inventory for Adults (HHIA) Questionnaire

The HHIA ( Table 5 ) was translated and adapted to Portuguese by Almeida (1998). 7 It is a self-assessment questionnaire of auditory handicap (hearing impairment), consisting of 25 items, of which 13 involve emotional aspects (E) and 12 involve social and situational aspects (S). Faced with each item or situation mentioned, the subject gave one of the following answers: 'yes' (4 points),'sometimes' (2 points) or 'no' (0 points). In the presence of doubts regarding the understanding of the question presented, clarifications were provided, being careful not to induce the patient's response to avoid causing measurement bias in the method.

**Table 5:** Hearing Handicap Inventory Questionnaire for Adults

	HEARING HANDICAP INVENTORY FOR ADULTS (HHIA) NAME: DATE:			
		**YES (4)**	**SOME- TIMES (2)**	**NO (0)**
S-1.	Does a hearing problem cause you to use the phone less often than you would like?			
E-2.	Does a hearing problem cause you to feel embarrassed when meeting new people?			
S-3.	Does a hearing problem cause you to avoid groups of people?			
E-4.	Does a hearing problem make you irritable?			
E-5.	Does a hearing problem cause you to feel frustrated when talking to members of your family?			
S-6.	Does a hearing problem cause you difficulty when attending a party?			
S-7.	Does a hearing problem cause you difficulty hearing/understanding coworkers, clients, or customers?			
E-8.	Do you feel handicapped by a hearing problem?			
S-9.	Does a hearing problem cause you difficulty when visiting friends, relatives, or neighbors?			
E-10.	Does a hearing problem cause you to feel frustrated when talking to coworkers, clients, or customers?			
S-11.	Does a hearing problem cause you difficulty in the movies or theater?			
E-12.	Does a hearing problem cause you to be nervous?			
S-13.	Does a hearing problem cause you to visit friends, relatives, or neighbors less often than you would like?			
E-14.	Does a hearing problem cause you to have arguments with family members?			
S-15.	Does a hearing problem cause you difficulty when listening to TV or radio?			
S-16.	Does a hearing problem cause you to go shopping less often than you would like?			
E-17.	Does any problem or difficulty with your hearing upset you at all?			
E-18.	Does a hearing problem cause you to want to be by yourself?			
		**YES (4)**	**SOME- TIMES (2)**	**NO (0)**
S-19.	Does a hearing problem cause you to talk to family members less often than you would like?			
E-20.	Do you feel that any difficulty with your hearing limits or hampers your personal or social life?			
S-21.	Does a hearing problem cause you difficulty when in a restaurant with relatives or friends?			
E-22.	Does a hearing problem cause you to feel depressed?			
S-23.	Does a hearing problem cause you to listen to TV or the radio less often than you would like?			
E-24.	Does a hearing problem cause you to feel uncomfortable when talking to friends?			
E-25.	Does a hearing problem cause you to feel left out when you are with a group of people?			

INSTRUCTIONS: The purpose of the scale is to identify the problems your hearing loss may be causing you. Check YES, SOMETIMES, or NO for each question. DO NOT skip a question if you avoid a situation because of your hearing problem.

( C W Newman , B E Weinstein , G P Jacobson , G A Hug 1990).

(Adaptation for Brazilian Portuguese- Almeida 1998).

No = 0 points Sometimes = 2 points Yes = 4 points.

Total of points / 100.

Total of points for SOCIAL / 48.

Total of points for EMOTIONAL / 52.

0–16% = No handicap.

18–42% = Mild-Moderate Handicap.

44%+ = Significant Handicap.

The score values can have variable percentage indices from zero to 100, with a correlation between the perception of the handicap and the score obtained. The high score suggests a significant perception of hearing impairment by the subject evaluated. Thus, a score from zero to 16 indicates no handicap; from 18 to 30, mild handicap; from 32 to 42, moderate handicap; and above 44, significant handicap.

## Results

The data obtained are presented below, according to the analyses performed for each evaluated questionnaire.

### 1-Analysis of the Results of the WHOQOL-BREF Questionnaire

The WHOQOL-BREF questionnaire consists of 26 questions (Q) divided into 4 domains: physical (7 questions), psychological (6 questions), social relationships (3 questions), and environment (8 questions).

**Fig. 1:**
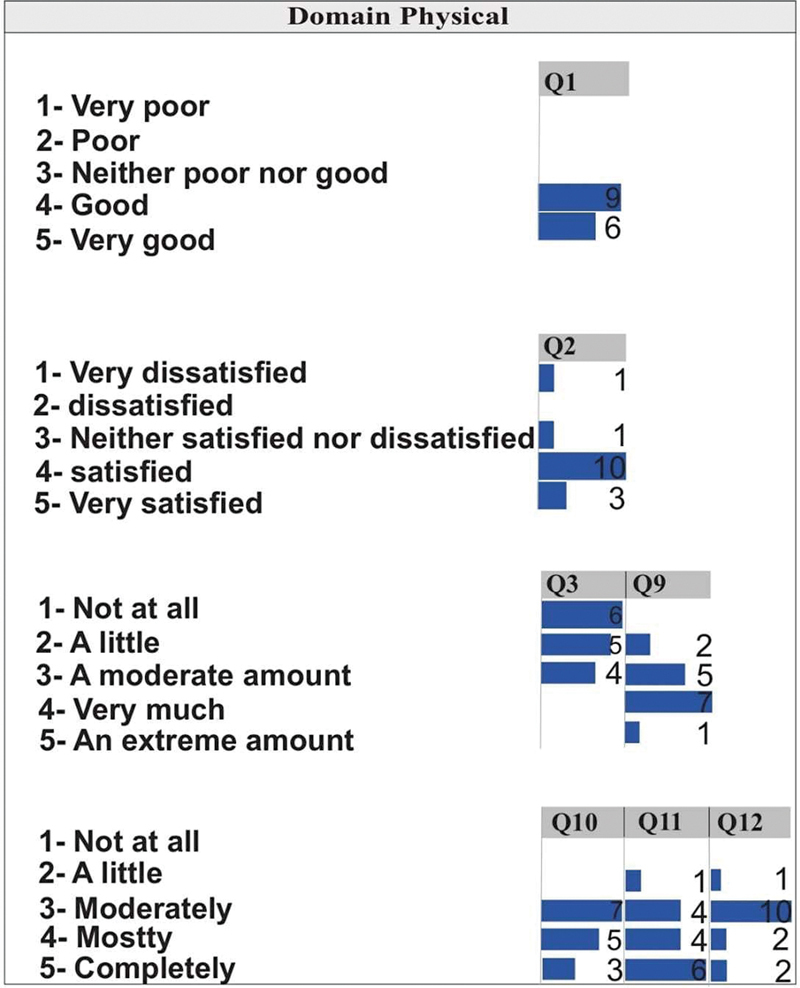
Quality of Life- Physical Domain.

Fig. 1 shows that for Q1, the patients evaluated the QoL as good or very good. It is observed for Q2 that most patients are satisfied with their health, one patient is dissatisfied, and one patient is indifferent.

For Q3, which assesses how much physical pain prevents exercise, it is observed that 6 of the patients report that the pain does not hinder anything, 5 that the pain interferes very little and 4 more or less. For Q9, which evaluates how healthy the physical environment is, 7 of the patients state that the environment is quite healthy, and only 1 reported it as extremely healthy.

Fig. 1 shows that in Q10 and Q12, which assess whether the patient has energy for the day to day and money to meet his or her needs, the patients were mostly in the medium category followed by the completely category. Q11 evaluates the acceptance of physical appearance. In this question, 6 of the patients said they were completely satisfied, and only 1 patient was a little satisfied.

**Fig. 2:**
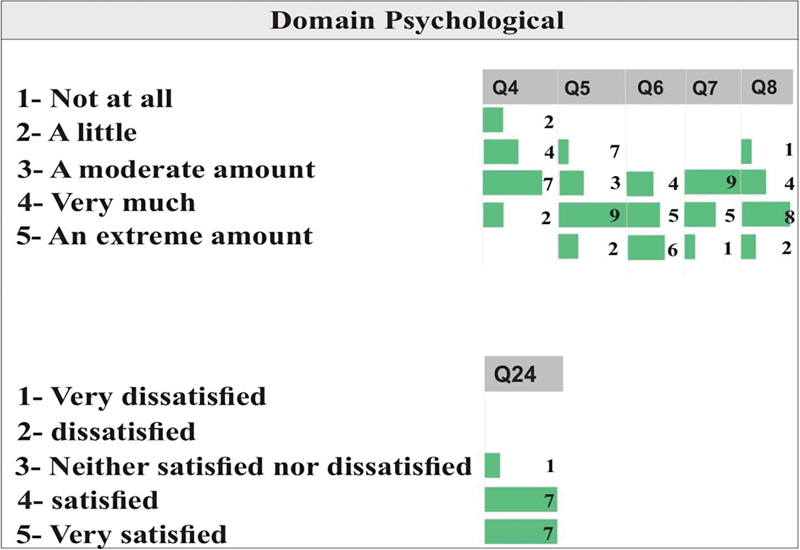
Quality of Life- Psychological Domain.

Fig. 2 shows that for questions Q4 and Q7, about the need for daily treatment and concentration level, the majority of patients said their needs were moderate. In Q6, 6 of the patients rated the meaning of life as “extremely” important, 5 as “very much” and 4 as “moderate.”

In response to questions Q5 and Q8, 9 and 8 of the 15 patients evaluated how much they enjoy life and how safe they feel in daily life as “very much.”

On Q24, the patients were satisfied or very satisfied with the health services, and only one patient was indifferent.

**Fig. 3:**
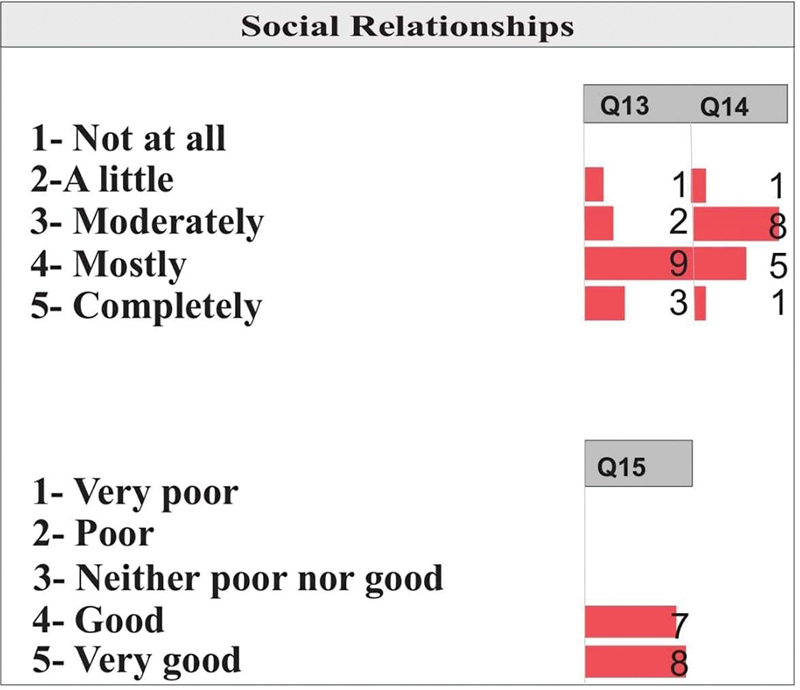
Quality of Life- Social Relationships Domain.

Fig. 3 shows that for Q13, most patients evaluated the availability of information as mostly available, followed by 3 patients who evaluated it as completely available. For Q14, 8 of the patients said they had moderate opportunities for leisure activity, followed by 5 patients who said they mostly had opportunities. Conversely, in Q15, 8 of the patients rated their walking ability as very good, followed by 7 patients who rated their ability as good.

**Fig. 4:**
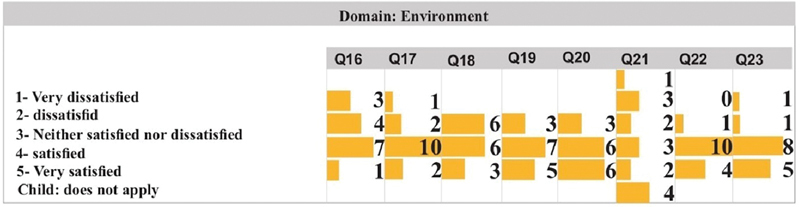
Quality of Life- Environment Domain.

Fig. 4 shows that in questions Q16, Q17, Q19, Q22, and Q23, the category most chosen by patients was satisfied. Q18 assesses satisfaction with work ability, and patients were indifferent, satisfied or very satisfied. In Q20, where satisfaction with personal relationships was evaluated, 6 of the patients rated themselves as very satisfied, 6 as satisfied and 3 as indifferent. In Q21, patients' answers varied more; 4 of the 15 patients did not answer because they were children, and the other patients were satisfied or dissatisfied.

**Fig. 5:**
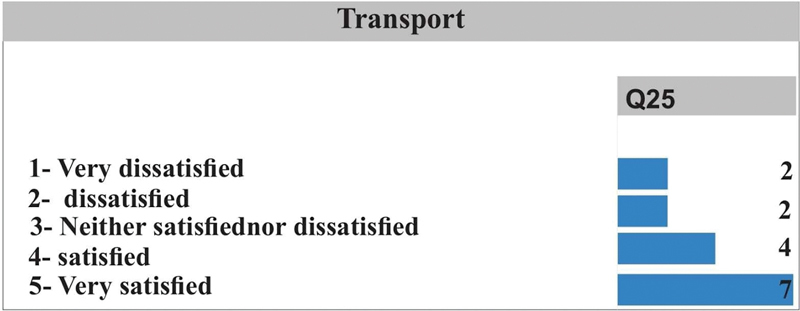
Quality of Life- Transport.

Fig. 5 shows that in Q25, the patients were mostly very satisfied with their means of transport, followed by 4 patients who said they were satisfied.

**Fig. 6:**
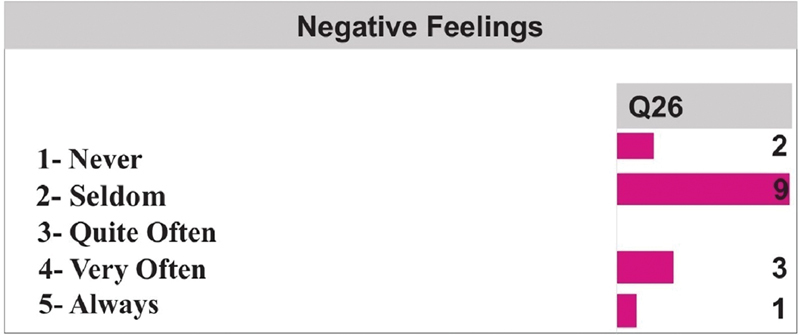
Quality of Life- Negative Feelings.

In turn, in Q26 ( Fig. 6 ), where the occurrence of negative feelings was evaluated, 9 of the patients stated that they occurred seldom, 3 patients said they occurred very often, 2 said never, and only one said always.

The results of the WHOQOL-BREF showed that the study participants classified their QoL as good or very good. In all questions, the vast majority reported being satisfied with the QoL, both emotionally and socially.

### 2-Analysis of the Results of the SSQ-12 Questionnaire

**Fig. 7:**
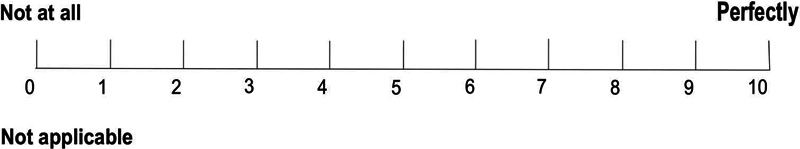
SSQ12 Scale.

The SSQ-12 Speech, Spatial and Qualities of Hearing Scale questionnaire consists of 12 questions to be answered considering the scale shown in Fig. 7 .

**Fig. 8:**
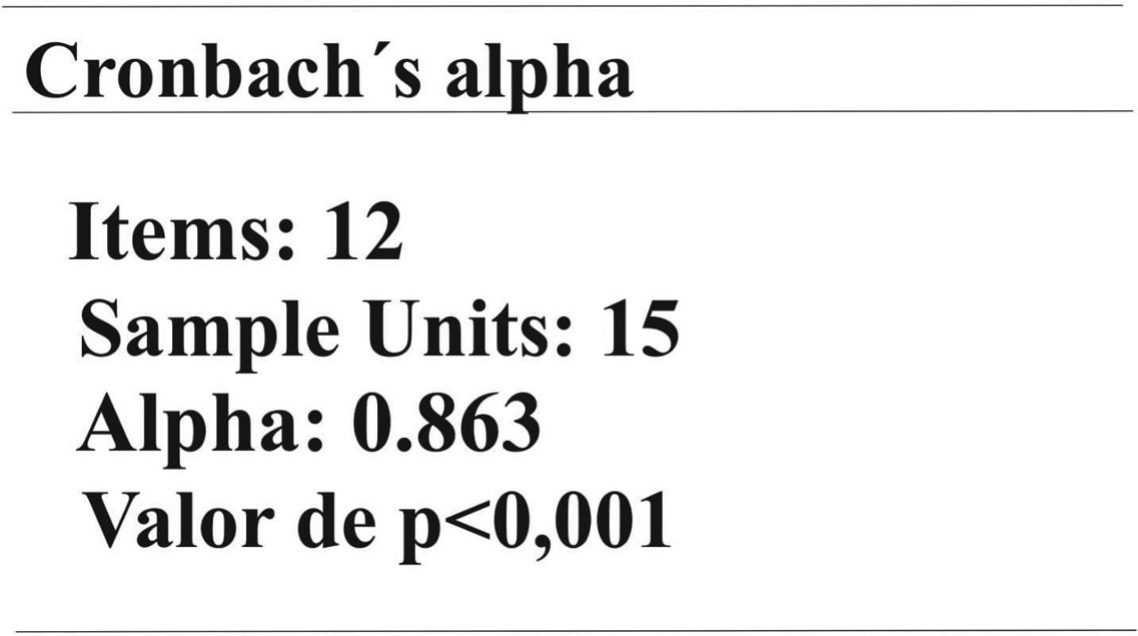
Cronbach alpha.

To evaluate the reliability and internal consistency of the questionnaire as a measuring instrument, Cronbach α coefficient was used, as its value was > 0.7 ( Fig. 8 ). There is evidence that the research or test items measure the same skill or trait. That is, the instrument is valid.

The SSQ-12 score ranges from 0 to 10, and the higher the score was, the better the auditory perception of the patient. Table 6 shows that the overall mean score was > 5.

**Table 6:** Descriptive Statistic for the answers to the questions - SSQ12 Questionnaire

	Statistics	Age (years old)	Interval Between Implants (years)	Q1	Q2	Q3	Q4	Q5	Q6	Q7	Q8	Q9	Q10	Q11	Q12
***n *** **= 15**															
	**Mean**	30.4	9.3	7.0	5.0	6.1	6.0	5.3	6.8	7.1	7.0	5.3	6.5	7.8	6.1
	**SD**	16.9	4.3	2.6	2.7	2.5	2.1	2.2	2.6	1.9	3.3	2.6	2.7	2.8	2.6
	**Minimum**	8.0	4.0	2.0	0.0	2.0	3.0	2.0	0.0	4.0	0.0	0.0	2.0	2.0	2.0
	**Maximum**	70.0	19.0	10.0	10.0	10.0	10.0	10.0	10.0	10.0	10.0	10.0	10.0	10.0	10.0
	**CV**	55.5%	46.5%	36.6%	53.5%	42.0%	34.5%	41.5%	38.2%	26.9%	47.4%	49.4%	41.7%	35.8%	42.2%

Abbreviations: CV, coefficient of variation; SD, Standard Deviation.

The mean score of each patient can be evaluated in Table 7 , considering age at the 1 ^st^ and 2 ^nd^ CI, as well as the use of hearing aids before CI. Patients with a mean score < than 5, a total of 3 patients started using late hearing aids and also underwent late cochlear implantation. Therefore, the positive result of the SSQ-12 shows that the participants report good auditory perception in various situations, even with an interval ≥ than 4 years between CI surgeries.

**Table 7:** Descriptive Statistic of SSQ12 X HHIA Scores

Age (Years old)	Age at 1st CI (Years old)	Time interval between implants (Years)	Age at 2nd CI (Years old)	Time of hearing aid use before CI (Years)	HHIA - S (Mean)	HHIA – E (Mean)	HHIA TOTAL (Mean)	SSQ12 (Mean)
8	3 (RE)	4	7 (RE)	2 (LE) 6 (RE)	20	0	20	5,08
13	1 (LE)	10	11 (LE)	0 (RE)11 (LE)	14	16	30	6,58
16	3 (RE)	10	13 (LE)	1 (RE) 11(LE)	30	38	68	6,83
17	5 (RE)	11	16 (LE)	1 (RE) 12 (LE)	18	32	50	4.08
19	1 (RE)	15	16 (LE)	1 (RE) 15 (LE)	8	14	22	7
26	2 (RE)	19	21 (LE)	19 (LE) 0 (RE)	2	0	2	9,41
26	4 (RE)	11	15 (LE)	3 (RE) 14 (LE)	26	32	58	7,56
26	16 (LE)	4	20 (RE)	14 (LE) 18 (RE)	10	12	22	5,58
30	17 (RE)	9	26 (LE)	13 (RE) 22 (LE)	42	34	76	3,33
32	23 (RE)	5	28 (LE)	16 (RE) 21 (LE)	14	24	38	7,66
34	15 (LE)	16	31 (RE)	9 (LE) 25 (RE)	28	30	58	8
37	30 (LE)	4	34 (RE)	22 (LE) 26 (RE)	44	50	94	4,16
43	22 (LE)	10	32 (RE)	16 (LE) 26 (RE)	16	22	38	6,91
59	41 (LE)	10	51 (RE)	10 (LE) 20 (RE)	6	10	16	7,25
70	62 (LE)	4	66 (RE)	32 (LE) 36 (RE)	20	4	24	6,58

Abbreviations: CI, Cochlear Implant(s); HHIA- E, HHIA Emotional; HHIA- S, HHIA Social; LE, Left Ear; RE, Right Ear.

### 3 - Analysis of the Results of the HHIA Questionnaire

The HHIA questionnaire consists of 25 questions divided into 2 subscales: emotional (13 questions that assess an individual's emotional behavior in relation to hearing loss) and social (12 questions that estimate the impact of hearing loss in several social situations).

For each question, there are three possible answers: 'yes' (4 points),'sometimes' (2 points) and 'no' (0 points). Based on the results, the emotional and social subscales were calculated separately. The sum of the points for the 25 questions was the total score.

The HHIA score can vary between 0 and 100; the social scale can range from 0 to 48, and the emotional scale can range from 0 to 52. Higher values indicate greater perception of hearing loss. Based on the score obtained in the questionnaire, the patient is classified according to the degree of hearing impairment according to the following criteria: score of 0–16 = no disability, 18–42 = medium disability, and 44+ = high disability.

**Fig. 9:**
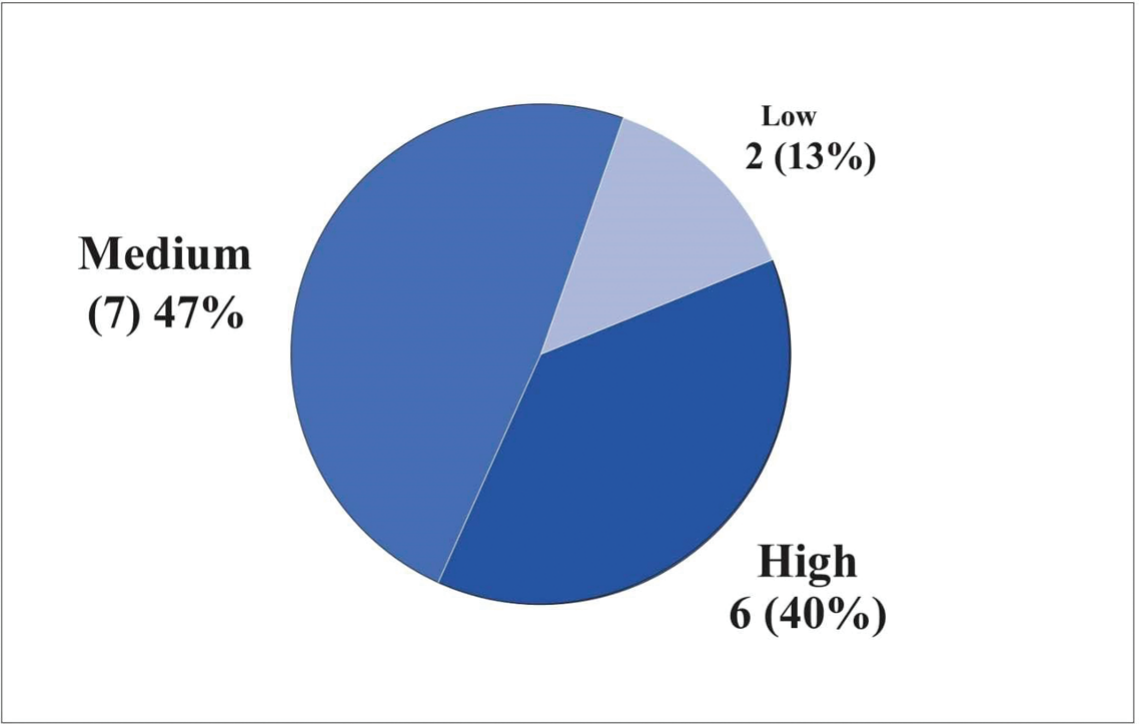
Distribution of Patients by degree of handicap.

According to the scores obtained in the questionnaires, 40% of the patients were classified as having a high degree of hearing impairment, 47% as having a medium degree and 13% as having a low degree ( Fig. 9 ).

**Fig. 10:**
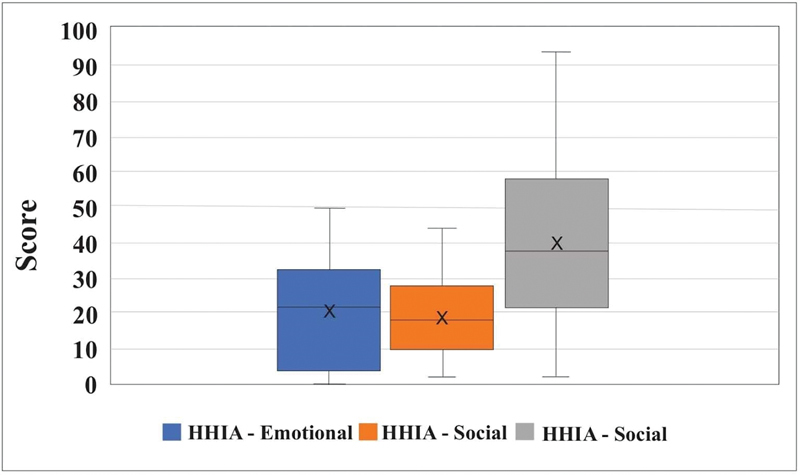
Distribution of HHIA Scores.

Fig. 10 and Table 8 shows that the mean scores and standard deviation (SD) values obtained in the HHIA questionnaire were equal to 40.4 ± 25.7 (total), 19.6 ± 11.8 (social), and 20.8 ± 15.2 (emotional).

**Table 8:** Descriptive Statistic of HHIA Scores

	Statistics	HHIA - Emotional	HHIA - Social	HHIA - Total
***n *** **= 15**				
	**Mean**	20.8	19.6	40.4
	**SD**	15.2	11.8	25.7
	**Minimum**	0.0	2.0	2.0
	**Maximum**	50.0	44.0	94.0
	**CV**	73.2%	60.3%	63.5%

Abbreviations: CV, coefficient of variation; SD, Standard Deviation.

The internal consistency of the HHIA using Cronbach α was 0.92 (total score), 0.86 (social) and 0.94 (emotional). Given that the α was > 0.7, there is evidence that the survey or test items measure the same skill or characteristic. That is, the instrument is valid. The Pearson correlation coefficient and the level of significance between the total score and the social and emotional subscales are shown in Table 9 .

**Table 9:** Pearson Correlation Coefficient

	HHIA - Social	HHIA - Total
HHIA - Emotional	r = 0.80	r = 0.96
	*p* < 0.001	*p* < 0.001
HHIA - Social		r = 0.93
		*p* < 0.001

**Fig. 11:**
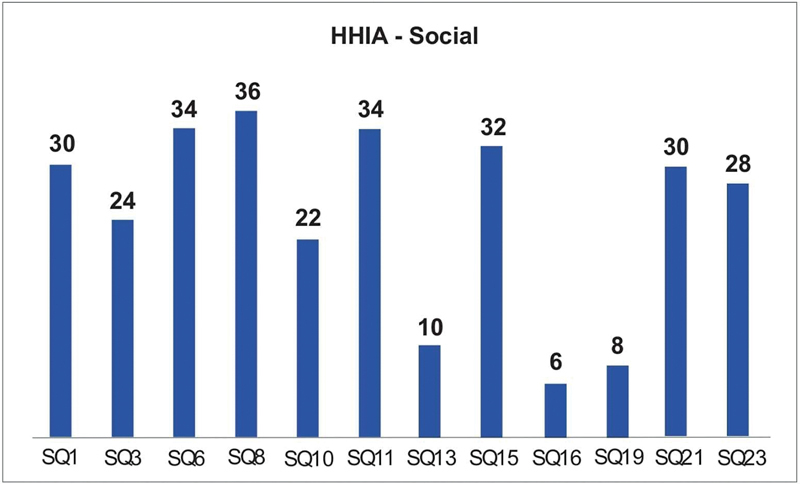
Score by Question HHIA-Social.

Fig. 11 shows that in relation to the social dimension, the question SQ8 (Do you have difficulty hearing when you go to the cinema or theater?) had a higher score and SQ16 (The difficulty in hearing makes you go out shopping less often. than you would like?) had the lowest.

**Fig. 12:**
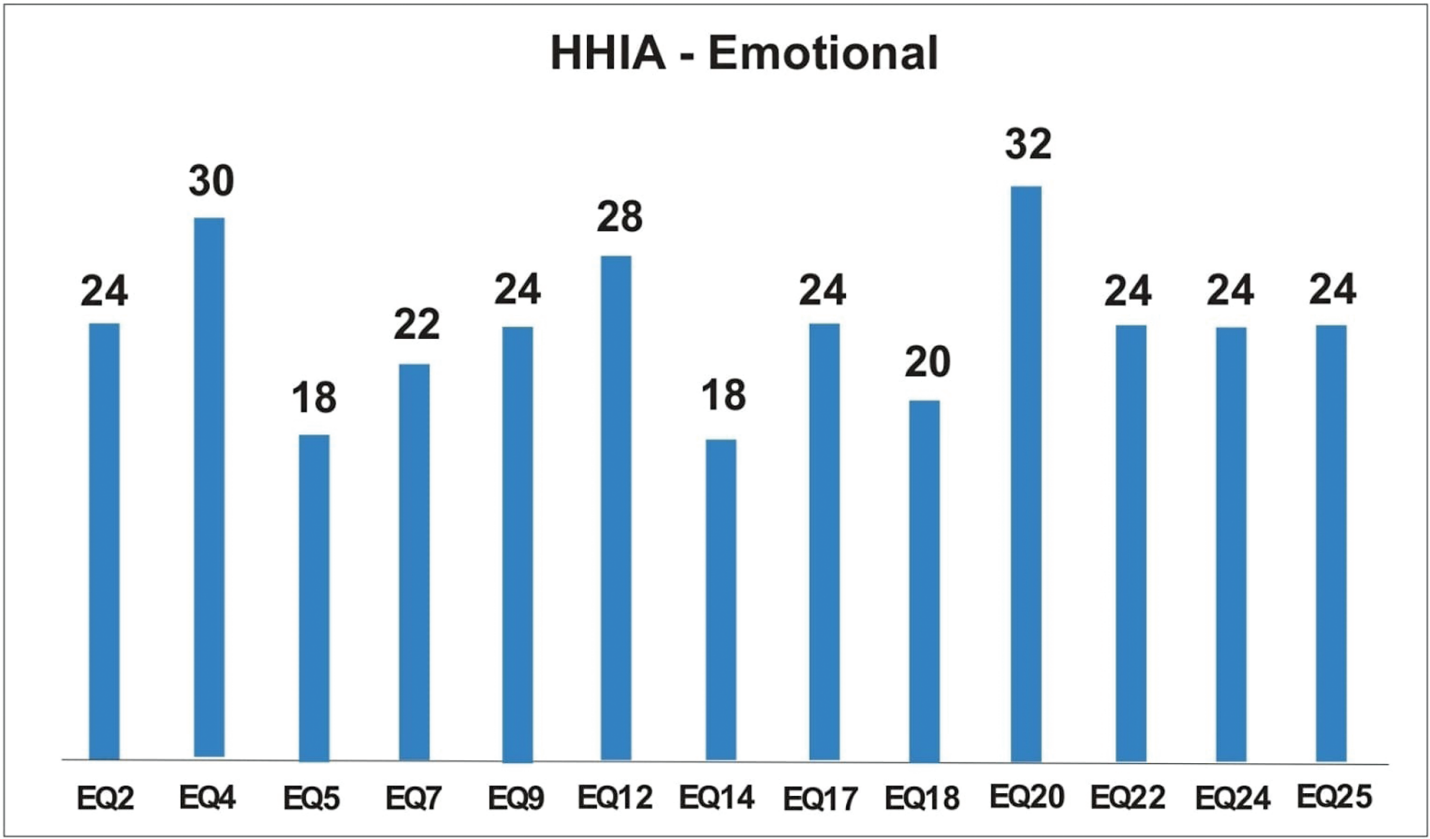
Score by Question HHIA-Emotional.

Fig. 12 shows that in relation to the Emotional dimension, the Eq. 20 question had the highest score and the Eq. 5 and Eq. 14 questions had the lowest scores.

The mean score of the HHIA in the study was 40.4, therefore classified as average disability with similar results in the emotional (20.8) and social (19.6) dimensions. According to the questionnaire, most individuals report that hearing loss somehow limits personal or social life but does not cause frustration or interfere with the activities of daily living (ADL).

## Discussion

In the previous literature, studies referring to QoL are qualitative studies or satisfaction questionnaires. Newman et al. 8 developed the HHIA by modifying the Hearing Handicap Inventory for the Elderly (HHIE) for individuals < 65 years old but maintained the self-assessment scale of patients with hearing loss, using 25 questions to address social and emotional issues.

In 2004, Gatehouseet al. 9 designed the Speech, Spatial and Qualities of Hearing Scale (SSQ-49), with 49 questions organized into 3 domains: hearing for speech, spatial hearing, and hearing quality. Aimed at rapid assessments that facilitate the care of hearing impairment in the clinical routine, an abbreviated version of the SSQ with 12 items was proposed, the SSQ-12, 10 derived from experiences in the use of the full-scale SSQ-49. The studies conducted with the reduced version showed that the results obtained are in close agreement with the average performance of the SSQ-49. 11

The QoL group of the mental health division of the WHO defined quality of life as the individual's perception of their position in life in the context of the culture and value system in which they live and in relation to its objectives, expectations, standards and concerns. 12 Two parameters are cited as important to the concept of QoL. One of them is subjectivity, which is to consider the person's perception of their health status, that is, how the person evaluates their personal situation in each dimension related to quality of life. 13 The other aspect is multidimensionality, which is the recognition that quality of life is linked to different dimensions. In this definition, the WHO takes as a basis a multifactorial nature of quality of life, considering six domains that aim to demonstrate the different dimensions of the human being in determining the levels of quality of life of each individual. Domains are categorized into physical health, psychological health, level of independence, social relationships, environment, and spiritual pattern.

First, the 100-question questionnaire known as the WHOQOL-100 was developed, 14 and to provide the application of the questionnaire in less time but have satisfactory psychometric characteristics, the WHO developed an abbreviated version: the WHOQOL-BREF. 15

Skevington et al. 16 evaluated the psychometric properties of the WHOQOL-BREF in adults from 23 countries. The economic variables, as well as health and rehabilitation conditions, were considered. The analyses indicated that the WHOQOL-BREF has great psychometric properties. For Seidl et al., 6 the WHOQOL-BREF is one of the most appropriate instruments to assess QoL because it considers the subjectivity and multidimensionality that make up the lives of people.

In the present study, the WHOQOL-BREF evaluation shows little discrepancy between individual responses, with a good portion of the participants claiming good quality of life in all domains tested. Even with the majority (47%) presenting a mean handicap in the HHIA, there is no evidence of communication disability according to the results in the SSQ-12, and everyday tasks are not made impossible by hearing impairment. However, individuals report having difficulty accompanying different simultaneous sounds, and hearing loss is a limiting factor of personal and social life.

Cochlear implants have become an effective and common treatment for people with profound hearing loss, and several studies have shown that users of this device can achieve significant improvements in their hearing skills. 17 18 19

Even though there are limiting factors in the daily lives of bilateral CI users, 13 individuals in the present study were satisfied with their health and most claimed to be satisfied with themselves and their ability to perform ADL.

Studies involving CI users over the years have analyzed the benefits of bilateral CI. According to Nelson et al., 20 speech recognition in the presence of competitive noise with unilateral CI is limited and suffers a negative impact when the spectral resolution is reduced.

Galvin et al. 21 investigated whether children subjected to sequential bilateral CI after a long surgical interval would have better benefits in auditory speech perception than those implanted unilaterally after 12 months of experience of bilateral CI. The authors found no significant differences regarding sound localization but reported benefits of auditory speech perception with bilateral CI for some of the evaluated individuals.

In a systematic review of the literature to evaluate the effect of the interval between sequential bilateral CI surgeries in adults and children, Smulders et al. 22 concluded that evidence from the literature suggested that the second CI can bring benefits of auditory speech perception, even after a substantial interval between surgeries in children, adolescents, and adults.

Friedmann et al. 23 emphasized that sequential bilateral CI should be considered, even after a long period of sensory deprivation in the second implanted ear and with a prolonged interval between CI surgeries.

Questionnaires that assess QoL and the limitations caused by hearing loss are widely used in the literature. Sparreboom et al. 24 evaluated the effect of sequential bilateral CI in children on their QoL. Six questionnaires were used to measure QoL: the Health Utilities Index Mark 3 (HUI3); the Pediatric Quality of Life Inventory (PedsQL); the Glasgow Children's Benefit Inventory (GCBI); the Speech, Spatial, and Qualities of Hearing Scale (SSQ); and the Nijmegen Cochlear Implant Questionnaire (NCIQ). The conclusion was that sequential bilateral CI in children is associated with an improvement in QoL.

In 2021, Sivonen et al. 25 studied the QoL and improvement in hearing after sequential bilateral CI in 27 adult patients with severe to profound hearing loss. The average interval between CI surgeries was 5.4 years, and of the 27 patients, only 8 were ISAD users before the second CI. The Glasgow Benefit Inventory (GBI) and Glasgow Health Status Inventory (GHSI) questionnaires were used to measure the impact of the second CI on QOL. Both questionnaires showed significant improvements after the second CI.

Sood et al., 26 in 2022, investigated the impact of unilateral sensorineural hearing loss (USNHL) on the QoL of patients using a validated questionnaire-Hearing Handicap Inventory for Adults (HHIA). The authors concluded that early diagnosis and rehabilitation are essential to prevent disability and raise QOL in patients with USNHL.

The present study, in agreement with previous studies, demonstrates that there is an improvement in the quality of life with the second CI. The evaluation of the results in CI programs is fundamental to measure the quality indicators and encourage the multidisciplinary team to offer an ethical commitment to the patient.

In this context, quality of life is an important aspect to be evaluated as a result of CI. The concern with assessing QoL goes beyond offering specialized technical knowledge. It is also necessary to include physical, emotional, environmental, and social factors, which characterize the intervention as humanized care.

Quantifying quality of life is essential to assess whether improving health contributes to improving QoL and whether improving QoL also improves health. In this sense, it is possible that CIs may favor the improvement of the QoL of patients with severe to profound hearing impairment because this device enables the improvement in auditory and language skills, especially with the advancement of new speech coding strategies. 27 28 The improvement in these skills is an important factor for communication and participation of these people in social life activities.

The evaluation of QoL using questionnaires directed at patients with hearing impairment is a valuable tool to measure adaptation to CI. It has already been proven by the scientific literature that the less time of hearing deprivation an individual endures, the better the response to auditory rehabilitation. However, we should not underestimate the improvement in hearing and QoL of patients who did not experience early hearing rehabilitation.

More studies evaluating the QoL of CI users are needed, as CI users still report hearing difficulty in the presence of distinct simultaneous sounds, and the use of questionnaires with self-assessment of patients is of great value to the scientific literature.

## Conclusion

In the present study, 15 individuals subjected to sequential bilateral CI were evaluated using questionnaires, with an interval ≥ 4 years between surgeries, and most reported good QoL and auditory perception.

Despite the limitations inherent to hearing loss and the postimplantation auditory rehabilitation process, the participants were satisfied with the sequential bilateral CI, according to the questionnaires applied in the study.
